# Fast Modeling of Binding Affinities by Means of Superposing Significant Interaction Rules (SSIR) Method

**DOI:** 10.3390/ijms17060827

**Published:** 2016-05-26

**Authors:** Emili Besalú

**Affiliations:** Institut de Química Computacional i Catàlisi (IQCC) and Departament de Química, Universitat de Girona, 17071 Girona, Catalonia, Spain; emili.besalu@udg.edu; Tel.: +34-972-41-8357

**Keywords:** SSIR method, analogue series, balanced leave-two-out (BL2O) cross-validation, ranking, SAR, inverse SAR

## Abstract

The Superposing Significant Interaction Rules (SSIR) method is described. It is a general combinatorial and symbolic procedure able to rank compounds belonging to combinatorial analogue series. The procedure generates structure-activity relationship (SAR) models and also serves as an inverse SAR tool. The method is fast and can deal with large databases. SSIR operates from statistical significances calculated from the available library of compounds and according to the previously attached molecular labels of interest or non-interest. The required symbolic codification allows dealing with almost any combinatorial data set, even in a confidential manner, if desired. The application example categorizes molecules as binding or non-binding, and consensus ranking SAR models are generated from training and two distinct cross-validation methods: leave-one-out and balanced leave-two-out (BL2O), the latter being suited for the treatment of binary properties.

## 1. Introduction

Methods exist to mine data of analogue series or combinatorial data sets, for instance, those based on SAR maps [1,2] or R-group polymorphisms [3], among others [4,5]. However, none is as simple as the Superposing Significant Interaction Rules (SSIR) method, a new systematic procedure able to rank analogue series that, in turn, constitutes an inverse SAR tool. The SSIR ideas was originated after the recent experience in a Design of Experiments (DoE) context treating molecular families sharing a common scaffold [6,7,8].

The SSIR method conceptualizes a combinatorial family as a series of sites (factors in DoE vocabulary [9]), each one having the ability to accommodate one of a set of various residues (levels). From this knowledge, combination rules of presence/absence of certain residues in sites are categorized as being significant or not. The SAR model consists of all the rules being categorized as significant. Each rule grants an additional positive or negative vote to each molecule that matches it. Hence, each analogue collects a series of signed votes that, once added up, establish a molecular ranking scale. It is expected that the ranked molecular series will correlate with the interest/non-interest molecular tags attached to the molecules according to the values of the analyzed property.

## 2. Results and Discussion

As application, here it is presented an example of Binding Activities modeling. In reference [5] a method is presented for obtaining SAR relationships of analogue series based on the analysis of dual-activity difference maps. The authors present as an example the application over a set of 106 pyrrolidine bis-diketopiperazines tested against two formylpeptide receptors (FPR). Table 1 presents the substitution codifications along the four available molecular scaffold sites.

The details of both experimental endpoints can be found in the aforementioned reference. Following the notation of the original work, the binding activities (*Ki*) here will be denoted as FPR1 (related to antibacterial inflammation and malignant glioma cell metastasis) and FPR2 (associated with chronic inflammation in systemic amyloidosis, Alzheimer’s disease and prion diseases). The goal of Medina-Franco *et al.* [5] was to compare both properties by working with differences arising from molecular pairs and looking for activity switches (*i.e.*, specific substitutions that have opposite effects on the activity of the compounds against two biological targets) and selectivity switches (minor structural modifications that drastically invert the selectivity pattern of two compounds). Here, each single property will be modeled.

Table 2 shows the codified set of 106 compounds. The original compound formulations can be found in Table S1 of the original article’s [5] supporting information. It is worth noting that the SSIR method can be applied systematically without the need for special preparatory operations (other methods require molecular minimizations, alignments, descriptor calculations and so on). The method’s symbolic nature means it is only necessary to arbitrarily codify the molecular substituents and decide which analogues are declared as being of interest (see materials and methods section below). These characteristics allow the SSIR method to model sets confidentially by masking the original information or molecule codification.

**Figure 1:**
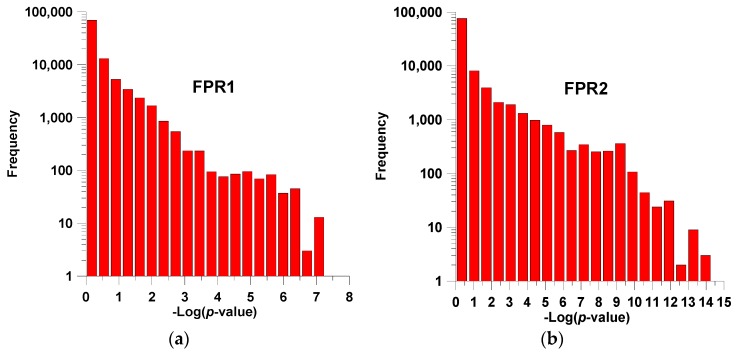
Distribution of *p*-values for all the definable rules of order 4 (negations allowed) for (**a**) FPR1; and (**b**) FPR2 properties. Note the logarithmic scale in both axes.

The library has four diversity points and the expanded set covers *M* = 5 × 8 × 9 × 17 = 6120 compounds. In the reference, *a* = 106 analogues are reported. In this set, the analogues of interest have been defined as those presenting low value of *Ki* expressed in terms of concentration in nM units. In both cases the *b* = 32 compounds (*ca.* 30%) presenting the lowest values were chosen as being of interest (property values lesser or equal to 411 and 410 nM for FPR1 and FPR2, respectively, and marked in Table 2 with asterisks in columns p*Ki*1 and p*Ki*2). The number of rules of order 1 (negations not allowed, see materials and methods section below) are 5 + 8 + 9 + 17 = 39. For orders 2–4 the number of rules are 531, 3029 and 6120, respectively. If negations are allowed, the numbers of possible rules increase to 78, 2124, 24232 and 97920, respectively. Figure 1 shows the distribution of *p*-values attached to the rules of order 4 (negation terms allowed) for both properties. It is noteworthy that property FPR2 reaches rules having much lower *p* values. This behavior is also found for other rule orders. The presence of more significant rules suggests that FPR2 could be better modeled.

### 2.1. Training and Cross-Validation

Table 3 shows the area under the receiver operating characteristic (*AU-ROC*) values [10,11,12,13] attached to the obtained ranking classification. Exploring the generation of rules of order 1, 2 and 3, immediate results were obtained for fitting and leave-one-out (L1O) tests. For all cases, the cutoff *p*-value was set to *p_c_* = 0.005. The total number of significant rules entering each calculation is given between brackets. Along the L1O or balanced leave-two-out (BL2O) cycles (see Section 3), certain rules present in fit are sometimes automatically discarded or some new significant rules appear as a result of the extraction and replacement steps. Hence, the total number of significant rules found along the cycles usually increases with respect to the single training calculation. Each BL2O calculation required 2368 cycles. In Table 3, the number of well classified pairs, ties and bad pair rankings encountered along the BL2O loops are explicitly indicated. For instance, regarding the FPR1 property, the BL2O involving rules of order 3 leads to 1909 well internally classified pairs, 2 ties and 457 incorrect pair rankings. For FPR2, the counts were 2253, 0 and 115, respectively. Those counts are related to *AU-ROC* values because it is well-known that, for a single fitting calculation, given a couple of molecules (one of interest and the other of non interest), the *AU-ROC* corresponds to the a posteriori probability that the classifier correctly sorts the pair [13].

In all cases, the second property is clearly modeled better by SSIR. As mentioned, this may be because the rules for FPR2 reach more significant (*i.e.*, small) *p*-values (see Figure 1). In other words, the analogues defined as of being of interest for FPR2 seem to be much more related to particular substituent combinations.

**Figure 2:**
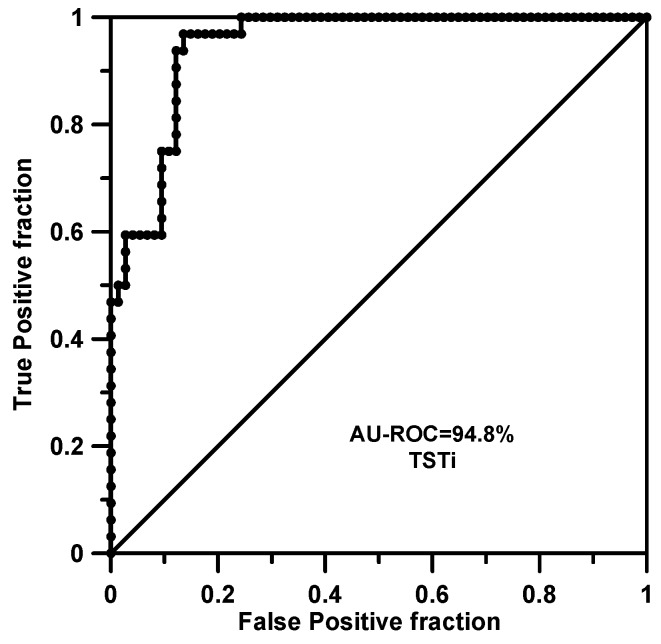
Receiver operating characteristic (ROC) curve and the area under it (*AU-ROC*) value for the FPR2 property calculated with the balanced leave-two-out (BL2O) cross-validation procedure (SSIR model involves rules of order 2, *p_c_* = 0.005).

Figure 2 shows the ROC curve for the BL2O ranking attached to the FPR2 property when rules of order 2 are being considered.

For this library, rules of order 2 are well suited to reveal general patterns attached to activity values of interest. Table 4 lists the first most significant rules of order 2 found for the FPR1 property. The systematic presence of G substituent becomes evident at position 2 (*S*-benzyl) attached to rules having a positive vote. Even more, the negation of G substituent at this position is systematically accompanied by a negative rule vote. The remaining rules mainly ask to avoid residue B (*R*-2-naphthylmethyl) at the same position. Other diverse combinations complete the full set of 117 selected rules having *p* ≤ 0.005. Inspection of the whole set of significant rules reveals that position 2 is the most relevant one when modeling the FPR1 property. This kind of information can be useful for some applications, for instance when a compound must be optimized in order to refine other molecular properties.

Table 5 lists the first most relevant rules of order 2 when modeling the FPR2 property. The pattern found in this list is the presence of residue C at the first substitution site (*S*-isopropyl). Again, the negation of residue C at this specific site is attached to a negative vote for the rules. Despite this particular rule behavior, molecular position 1 is not the most relevant one, as seen from an inspection of the full set of 447 selected rules. In this group of significant rules, one encounters a diversity of residues to be placed or avoided at specific positions. Hence, the FPR2 property is modeled from several “points of view” regarding combinations of substituents. This variety of choices confers better modeling options to the property and, in this case, a more robust final SSIR model.

**Figure 3:**
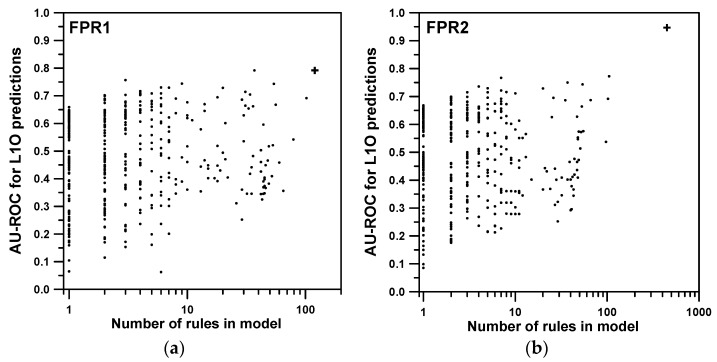
Randomization test leave-one-out (L1O) predictions of *AU-ROC* values that could be obtained for the (**a**) FPR1; and (**b**) FPR2 properties after 1000 cycles. Horizontal axes (logarithmic units) show the number of rules entering in each SSIR model.

The results in Table 3 have been checked by means of randomization tests. These tests consist of randomly scrambling all the molecules’ interest/non-interest labels and redoing the modeling calculations from scratch 1000 times, *i.e.*, generating all the rules again from the beginning and recalculating all the probabilistic *p* values. Figure 3 shows the fake *AU-ROC* values obtained for the FPR1 (Figure 3a) and FPR2 (Figure 3b) properties through L1O predictions. The calculation involves the rules of order 2 (*p_c_* = 0.005). During the cycles, a SSIR model could only be reproduced 428 (Figure 3a) or 409 (Figure 3b) times. For the other cases, all the rules’ significances were greater than the threshold *p_c_*. The “randomized” models are represented by a point in the graph whereas the correct model is represented by a cross. The points always present lesser *AU-ROC* values (vertical axis) than the correct model (except for a model for the FPR1 property). The graph also shows how the number of rules found per randomized test is lesser than 117 (Figure 3a) and 447 (Figure 3b), the number of rules defining the correct model. For FPR1 a fake model consisted of 102 rules and 105 for FPR2. All this data confirms again that the FPR2 property is much better modeled than FPR1, as the corresponding cross in Figure 3b is clearly further away from the cloud of randomized points.

Possibly, FPR1 property is being overparametrized as several *L1O-AU-ROC* values fall near the unscrambled test. It has to be noted that, in some cases, the random scrambling of molecular interest/non-interest tags leads to situations that can be partially modeled by SSIR. For instance, if the original tags pointing to molecules having low property values (analogues of interest) are mainly placed on molecules having higher property values during scrambling, then the situation becomes a sort of complementary version of the original one and SSIR is able to model it. Unfortunately, another undesirable possibility is left to chance: in some cases the random placement of the tags can set the analogues of interest in a partially correlated way with respect to one or more substituents. This situation will also be modeled by SSIR, generating fake rules. Therefore, the combination of both methodologies, cross-validation and randomization tests, are to be taken into account when modeling with SSIR in order to detect spurious models.

### 2.2. Inverse Structure-Activity Relationships (SAR): New Analogue Proposals

The SSIR method is an inverse SAR tool [14] because of its ability to suggest new compounds. For the model of rules of order 3 (*p_c_* = 0.005, negations allowed), the SSIR program was asked for predictions of new combinatorial analogues having a high number of positive votes. The SSIR program generated all the remaining 6014 items that were not training compounds. For this set of new analogues, the number of votes coming from the rules ranged from −376 to +397 for FPR1 and from −1101 to +1283 for FPR2. Each list provides a ranking attached to either property. The first ranked compounds in each list are the following: EGEN, EGEO, EGEL, EGEM, EGEI, EGEG, EGEE, EGED, EGEF, EGEB, EGEC, EGEQ, EGEA, DGEP, DGEK, DGEI, DGEE, DGEG, DGEQ, DGED, DGEB, DGEF, EGFJ, EGIJ, EGFH, EGIH, EGFK, DGEA, EGIK, EGGP, EGGJ, EGAP, EGAJ, EGFM, EGFN, EGFL, EGFO, EGIO, EGIL, EGIN, EGIM, EGFI, EGGH, EGGK, EGII, EGAH, EGFE, EGFG, EGAK (for FPR1); and CACD, CACE, CACF, CBCD, CACI, CACQ, CACL, CACM, CACK, CACG, CACN, CACO, CACB, CACP, CACJ, CBCI, CBCQ, CBCO, CBCK, CBCG, CBCM, CBCN, CBCL, CBCJ, CBCB, CBCP, CACA, CBCA, CADH, CAHH, CAIH, CABD, CABE, CABF, CABI, CABQ, CABN, CABL, CABM, CABO, CABK, CABB, CABJ, CABP, CABG, CAFH, CBDH, CADC, CAAD, CBBD (for FPR2).

In both complete lists, 190 compounds simultaneously present positive votes. For illustrative purposes, it is also possible to merge both lists and propose compounds presenting good expectations of being active for both properties at the same time. This was accomplished by linearly scaling the intervals of integer votes per property into a real interval. The negative votes have been scaled in the interval [−1,0) and the positive votes have been scaled in the interval (0,+1]. The real value 0 is reserved for the analogues having just zero votes. Then, for each compound, the new real scaled votes related to each property were added up. This sum serves as the new ranking parameter. The following is the list of the first 99 ranked analogues in decreasing order of preference: CGCH, CGCJ, CGCP, CGCO, CGCN, CGCM, CGCL, CGCK, CGCC, CGCD, CGAH, CGCI, CGCE, CGCF, CGCQ, CGCG, CGCB, CGIH, CGBH, CGAJ, CGAP, CGFH, CGCA, CGIJ, CGBJ, CGDH, CGAM, CGAN, CGAL, CGAO, CGAK, CGHH, CGIP, CGBP, CGAD, CGAC, CGAI, CGAE, CGAF, CGAQ, CGIM, CGIN, CGIO, CGIL, CGBO, CGBL, CGBM, CGBN, CGIK, CGAB, CGAG, CGBK, CGFJ, CGID, CGIC, CGII, CGIE, CGIF, CGBI, CGBD, CGIQ, CGBC, CGBE, CGBF, CGDJ, CGIG, CGIB, CGFP, CGBQ, CGHJ, CGDP, CGBB, CGBG, CGFN, CGFL, CGFO, CGFM, CGFK, CGDM, CGDO, CGDL, CGDN, CGHP, CGDK, CGFD, CGFI, CGDD, CGFC, CGFE, CGDE, CGDI, CGHN, CGHO, CGHM, CGHL, CGFQ, CGFF, CGDF, CGDC.

Another calculation was conducted to generate external analogues. To prevent extrapolations outside the training set chemical space, consideration was only given to compounds having at most one single substitution difference (in any site) respect to at least three training compounds (of course, other choices are possible). A total of 511 analogues fulfill this condition. For this set of new analogues, the number of collected votes ranged from −396 to +397 for FPR1 and from −1101 to +1278 for FPR2. The first 50 ranked compounds in each list are the following: EGEN, EGEO, EGEL, EGEM, EGEI, EGEG, EGEE, EGED, EGEF, EGEB, EGEC, EGEQ, DGEP, EGEA, DGEK, DGEI, DGEG, DGEE, DGEQ, DGED, DGEB, DGEF, EGFJ, DGEA, EGAP, EGGP, EGGH, EGBP, EGDP, DGGJ, EGHP, DGGP, DGFH, EGCP, DGGO, DGGN, DGGM, DGGL, DGGK, DGFC, DGGI, DGGE, DGGG, DGGD, DGGQ, DGGF, BGEJ, DGGB, DGFA, BGEP (for FPR1); and CACE, CACF, CBCD, CBCI, CBCQ, CBCN, CBCK, CBCM, CBCO, CBCG, CBCL, CBCJ, CBCB, CBCP, CACA, CBCA, CADH, CAHH, CAIH, CABD, CABB, CABG, CAFH, CBDH, CADC, CBBD, CBBE, CBBF, CBHH, CBBG, CBBB, CBIH, CAHC, CAIC, CBAD, CBDC, CBFH, CBAE, CBAF, CAFC, CCCH, CAAA, CBHC, CBIC, CFCH, CBFC, CBAA, CCCC, CFCC, CECH (for FPR2).

A total of 10 compounds have positive votes in both complete lists. The ranked list of scaled and added votes sorted the analogues as follows: CGEH, CGAH, CGAC, CGBH, CGBC, BGCH, BGCC, CGCH, CGCC, EDBH. Note that six compounds (underscored) are also present in the above list of 99 selected analogues. According to the general rules stated above (Table 4 and Table 5), most of the selected compounds present the *G* residue at position 2 and the *C* residue at position 1.

As presented, the lists of proposed molecules ranked by SSIR help to prioritize structures for synthesis, screening, database pruning or selection. As shown, the rules can be combined to detect common prioritized structures. This optimization task needs not be immediate or easy in general, especially in those cases where the multiple objectives are contradictory (negatively correlated). It is not the goal of this article to deal with the topic of multiobjective optimization [15] and the related issues will be published elsewhere.

### 2.3. Advantages and Drawbacks of the Method

One of the advantages of the SSIR method (described in Section 3) is that the input data can be prepared fast because pre-process tasks are minimal. Starting the process is immediate because conformational analysis, molecular superpositions and index calculations are not needed. The symbolic treatment can be interpreted as a sort of encryption. Hence, the modeling procedure can be offered to a third party in a confidential manner, *i.e.*, without revealing the molecular database being studied.

Of course, the method has drawbacks. Apart from those cited above, the molecular space couldn’t be explored beyond the substituents codified in the training database. This conditions the eventual test or validation molecular set structure (other methods, such as Inductive Logic Programming [16], allow the application of generated rules to molecules presenting new substituents). It is also worth mentioning that the results depend on the balancing of the database and on the degree of library dilution with respect to the full definable set of molecules. The method cannot deal with libraries of analogues presenting only one single substitution site. Our current research focuses on getting selected rules of higher order, as exhaustive generation is not possible in many cases due to combinatorial explosion. Work is also done to apply the method to continuous property values.

SSIR constitutes a rules search engine that has been presented here in a net context. The inner procedure can be improved, and additional benefits are attainable with the help of other techniques mainly devoted to rule management.

## 3. Material and Methods

### 3.1. Libraries, Sublibraries, Rules and Negation Terms

A congeneric molecular series sharing a common scaffold with *n* anchorage sites is visualized by the SSIR method as a structure having *n* factors. In turn, each substitution point *i* is able to accommodate *m_i_* residues or building blocks (relevant sites are only those for which *m_i_* > 1). In this manner the total number of analogues definable in the library is

(1) $$M=\prod_{i=1}^{n}m_{i}$$

and each analogue is identified by the list of sorted residues, for instance *A*_1_*B*_2_*B*_3_...*C_n_* or, simply, *ABB...C*, as the position of each letter specifies the substitution point.

The SSIR procedure assumes that the molecular property values depend on the effect of some relevant substituents placed at some relevant sites, but also that these non-linear effects can be expanded by superposing rules involving only a few sites.

**Figure 4:**
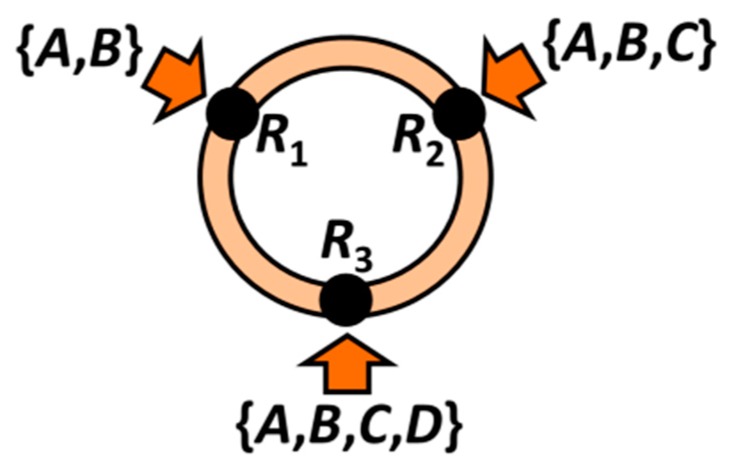
Representation of the toy model of molecular scaffolding having three substitution sites that admit 2, 3 and 4 residues, respectively.

For illustrative purposes, let us consider a toy library obtained by a combination of residues in 3 sites (see Figure 4). The full set of molecules belonging to the library is given by the Cartesian product *R* = *R*_1_ × *R*_2_ × *R*_3_ where the levels for each site are represented in turn by the site sets *R*_1_ = {*A*,*B*}, *R*_2_ = {*A*,*B*,*C*} and *R*_3_ = {*A*,*B*,*C*,*D*}. The symbols associated with each residue, despite being repeated, generally stand for distinct codified entities along the sites. If some residues are identical for two or more sites, the repeated notation always remains unambiguous because the corresponding influence over the molecular properties is distinct due to the positional effects. For this example, *m*_1_ = 2, *m*_2_ = 3 and *m*_3_ = 4. Hence, the complete library *R* contains *M* = 2 × 3 × 4 = 24 analogues collected in the universal set *R* = {*AAA*, *AAB*, *AAC*, ..., *BCC*, *BCD*}. It should be noted here that the entire library is generally not at the disposal of the researcher; usually only a fraction of it is known (*i.e.*, already synthesized and with the molecular property evaluated).

Here, the wildcard notation *X* will stand for any of the residues belonging to a particular anchorage point. Hence, the full database is also denoted by *R* = {*X*}_1_ × {*X*}_2_ × {*X*}_3_ = {*XXX*} or, simply, *XXX*. When applying the SSIR method, it is very important to define partial subsets of analogues taken from the library. For instance, the rule *XAX* stands for all the analogues presenting residue *A* at the second site. This is the same as in the molecular set

*XAX* = {*AAA*, *AAB*, *AAC*, *AAD*, *BAA*, *BAB*, *BAC*, *BAD*}

It is said that this rule embraces or condenses *m*_1_ × *m*_3_ = 8 analogues. Rule *XAX* is of order 1 because it establishes substitution restrictions in one site. This virtual library also admits rules of order 2 (the ones setting substitution restrictions in two sites, as for the case of *XAD* standing for the two analogues simultaneously presenting residues *A* and *B* at positions 2 and 3, respectively) or order 3 (such as the rule identified with the analogue *BBC*). The maximum rule order definable in a library is *n*, the number of substitution slots. The total number of rules (assuming that there are not redundant molecular symmetry issues) is

(2) $$V=\sum_{k=1}^{n}\left\{\sum_{i_{1}=1}^{n-k+1}\sum_{i_{2}=i_{1}+1}^{n-k+2}\ldots \sum_{i_{k}=i_{k-1}+1}^{n}\left(\prod_{j=1}^{k}m_{i_{j}}\right)\right\}$$

In Equation (2) the leftmost summation defines the rule orders, the inner *k* summation symbols constitute a Nested Summation Symbol (NSS) [17,18,19,20] and generates the combinations of *k* elements taken from the pool of *n* (the selection of sites involved in each rule) and the rightmost product counts how many rules are being generated from the previously selected *k* substitution sites. This last term corresponds to the combinatorial object variations with repetition. Given the identification of the sites being combined (values of *i*_1_, *i*_2_,...,*i_k_*), the number of generated rules of order *k* arising from it is

(3) $$v_{k}=v\left(i_{1},i_{2},\ldots ,i_{k}\right)=\prod_{j=1}^{k}m_{i_{j}}$$

The number of compounds being condensed by each rule of *k*-th order is

(4) $$c_{k}=c\left(i_{1},i_{2},\ldots ,i_{k}\right)=\prod_{j\neq i_{1},i_{2},\ldots ,i_{k}}^{n}m_{i_{j}}=\left(\prod_{j=1}^{n}m_{j}\right)/\left(\prod_{j=1}^{k}m_{i_{j}}\right)=\frac{M}{v_{k}}$$

Regarding the toy example above, Equation (2) generates a total of 9 rules of order 1 (2 + 3 + 4). The rules of order 2 arise from the combinations of sites 1–2, 1–3 and 2–3 and a total of 6 (2 × 3), 8 (2 × 4) and 12 (3 × 4) rules are generated, respectively. There are a total of 2 × 3 × 4=24 rules of order 3 (the maximum order definable in this example). Hence, the complete sum (2) is *V* = 59.

Equations (2)–(4) involve rules made with “positive terms” for which the presence of a residue is required. The universe of rules increases if rules involving the concept of 'non presence of a specific residue' are also taken into account. These are termed negation terms or negation operators. The negation operator of a certain residue is denoted by a bar as in rule $X\bar{A}X$, defining a complementary term, which here stands for the set {*XBX*, *XCX*} condensing the 2 × 1 × 4 + 2 × 1 × 4 = 16 analogues not having residue *A* at the second position. The combination of two complementary variables can lead to other specific molecular sets. Take as an example the rule

(5) $$\bar{A}\bar{B}X=\bar{A}XX\cap X\bar{B}X$$

which condenses the 8 analogues not having residue *A* at the first site and simultaneously not having the residue *B* at the second. That is the same as the set

{*B*} × {*A,C*} × {*A,B,C,D*} = {*B*} × {*A*,*C*} × {*X*} = {*BAX*, *BCX*}

Despite that algebra opens the possibility to define many combinations of negation terms along the rules (see Appendix), here all the rules will only involve juxtaposed positive (*A*, *B*, ...) or negative ($\bar{A}$,$\bar{B}$, ...) terms. A word of caution must be given here: it is not always necessary to attach negation operators at every site. It will be redundant to apply negation operators to binary sites (those presenting only two residues) because each level is the natural negation of the other. Specifically, for a binary site set {*A*,*B*} the complementary rule $\bar{A}$ is the same as the rule *B*. Conversely, the rule $\bar{B}$ is also equivalent to rule *A*.

All in all, systematic rule generation by computation is performed nesting three combinatorial entities: combinations among *k* sites, defining the rule order; the generation of variations among the residues attached to the previously selected sites; and another variational generator taking into account the presence or absence of individual negation terms (adding at most a 2*^k^* expanding term). References [17,18,19,20] provide information about how to implement these discrete algorithms.

### 3.2. Rules Significance and Votes

The relative relevance of a rule arises after a preliminary dichotomization of the library. The investigated molecular property must be codified in a binary fashion (in general tagging each analogue as being or not being of interest, *i.e.*, being or not being active, a drug, desirable, and so on). Many times the original property is not binary, and in these cases the SSIR method requires an arbitrary threshold frontier. Once the available molecular set presents only two classes, associating a significance *p*-value to each rule is immediate [21,22,23]. The calculation involves the number of molecules of interest which are present (known) in the library and are condensed by the rule: if the known sublibrary consists of *a* ≤ *M* analogues, being *b* of them declared of interest, a particular rule condensing *c* known molecules has the following hypergeometric probability to condense *d* out of *c* being also of interest:

(6) $$P\left(d,c;b,a\right)=\frac{\left(\begin{array}{c} b\\ d \end{array}\right)\left(\begin{array}{c} a-b\\ c-d \end{array}\right)}{\left(\begin{array}{c} a\\ c \end{array}\right)},d\leq c\leq a\leq M,d\leq b\leq a\leq M$$

The cumulated probabilities that the rule condenses *d* or more (*d*+) structures of interest define the significance level or *p*-value:

(7) $$p\left(d+,c;b,a\right)=p\left(d:min\left(b,c\right),c;b,a\right)=\sum_{i=d}^{min\left(b,c\right)}P\left(i,c;b,a\right)=1-\sum_{i=max\left(0,c+b-a\right)}^{d-1}P\left(i,c;b,a\right)$$

For instance, in our example above, let us assume that, from the *M* = 24 analogues, only *a* = 15 are at our disposal with known activity and that, from these, *b* = 5 are declared of interest. Then, if a rule such as *XAX* condenses *c* = 6 known molecules (the remaining 2 are not yet synthesized) and *d* = 4 of them are found to be active, expression Equation (7) indicates that the probability of randomly collecting 4 or more active molecules is *p*(4+,6;5,15) = 0.047. If this value is (arbitrarily) considered significant, the SSIR method will assume that rule *XAX* is a promising one, and will include it in the SAR model. Then, the assumption is that the 2 analogues that are not yet synthesized are expected to have promising chances of being active. The SSIR method assumes that the superposition of many significant rules provides a ranking method that increases the chances of pointing out new active analogues not present in the known sublibrary. This assumption requires an important aspect: establishing an arbitrary *p*-value cutoff (*p_c_*) defining “significant” rules (in our example we have assumed that *p_c_* = 0.05).

The SSIR SAR model is additional in the sense that each compound (being present or not in the known training library) is assigned to a number of votes coming from the significant rules that condense it. Besides, some rules entering the model have a negative vote. For instance, if the rule *BCX* significance is *p*(*d*+,*c*;*b*,*a*) > *p_c_*, then the SSIR protocol focuses the attention on the complementary probability counterpart and evaluates the event consisting of collecting *d* or less active compounds, *p*(*d*−,*c*;*b*,*a*). It is easy to demonstrate that

(8) *p*(*d*−,*c*;*b*,*a*) = 1 − *p*(*d*+,*c*;*b*,*a*) + *P*(*d*,*c*;*b*,*a*) = *p*([*b*−*d*]+,*a*−*c*;*b*,*a*)

If the condition *p*(*d*−,*c*;*b*,*a*) ≤ *p_c_* is satisfied, the rule *BCX* receives a negative vote, and this vote will be inherited by all the structures condensed by the rule. In fact, this procedure is equivalent to granting a positive vote to the full negation complementary rule $\bar{BCX}$.

It is assumed that the higher the number of net positive votes a structure collects, the higher the probability of it being of interest. Conversely, analogues attached to net negative votes are presumably of non-interest. The SSIR goal is to efficiently impute votes not only to training compounds but also to new test or validation analogues not present in the available sublibrary, which serves as a training set.

Once a training set library is given and the compounds of interest are defined, the our SSIR code generates rules, keeps the significant ones according to the pre-established cutoff *p*-value, and assigns positive or negative votes to them. The SSIR SAR model consists of the whole set of selected rules and attached votes. The model is then ready to be applied to a test or external validation set. Future investigations are to be conducted in order to establish the efficiency of SSIR relative to the known (synthesized) percentage of the library and also to the effect of the actual distribution of substituent combinations.

### 3.3. Cross-Validation

As a way to test the model’s predictive capabilities, several cross-validation procedures are implemented in our SSIR program code. Here the leave-one-out (L1O) and the balanced leave-two-out (BL2O) tests have been considered. Both tests are iterative and require the generation of predictions either for single analogues (L1O) or pairs of them (BL2O). All the L1O and BL2O cross-validation cycles have been designed according the Internal Test Sets (ITS) method [24,25,26] consisting of generating all the models from the beginning, *i.e.*, selecting all the rules from scratch as if the left out cross-validated analogue(s) were not present in the original library. This constitutes a realistic cross-validation simulation, which helps to detect overparameterization effects. Despite this being a time-consuming task, fortunately the accumulative nature of SSIR rules allows the final results to be obtained after fast reckoning procedures. For instance, in order to implement a L1O procedure it is only necessary to perform the first full training involving all the analogues and keep in disk or computer memory the number of relevant compounds condensed by each rule and the number of these which are of interest. Then, during the cross-validation cycles, for each virtually left out structure it is only necessary to do the simple re-count of condensed and active structures that would be obtained if the model were rebuilt from scratch without the cross-validated analogue. In essence, this is a way to see which effect produces the momentary absence of each cross-validated analogue among each rule. This allows immediate recalculation of the rule significances and reconsideration of the vote assignation.

The same applies for the BL2O cross-validation procedure. The method consists of looping over all the pairs of active and non-active analogues and cross-validating them. This prevents generating all the combinations of molecular pairs except *n_act_* × *n_nact_*, the product of the number of active (*n_act_*) analogues by the number of non-actives (*n_nact_*). For every left-out pair of molecules, two prediction votes are given and are added up. At the end of the full procedure, the sum of votes of the active and non-active compounds is divided by *n_nact_* and *n_act_*, respectively, giving a set of homogenate ranking votes. As for the L1O case, a fast version of the BL2O procedure has also been implemented, getting for each rule the new attached vote (if any) after carefully managing the respective condensation counts.

## 4. Conclusions

SSIR, a systematic procedure used to rank series of combinatorial analogues, has been described. In addition to this general description of the method, an illustrative application example has been provided. The method has been shown to be fast and systematic, leading to good predictions in some cases. Some overparametrization features can be easily detected relying on cross-validation or randomization test procedures. It has also been shown that the SSIR method constitutes an inverse (Q)SAR tool engine. The balanced leave-two-out (BL2O) cross-validation procedure has been also described.

## Figures and Tables

**Table 1:** Molecular substitution codifications. Note that each letter represents a distinct substituent depending on the substitution site.

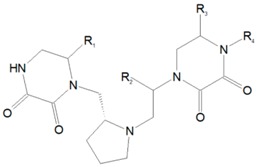

Code	R_1_	R_2_	R_3_	R_4_
A	*R*-2-naphthylmethyl	*R*-4-hydroxybenzyl	*R*-benzyl	4-Methyl-1-cyclohexyl-methyl
B	*S*-propyl	*R*-2-naphthylmethyl	*R*-4-hydroxybenzyl	Cyclohexylpropyl
C	*S*-isopropyl	*R*-cyclohexyl	*R*-butyl	Cyclohexylmethyl
D	*S*-butyl	*R*-propyl	*R*-propyl	Cyclopentylmethyl
E	*S*-benzyl	*S*-hydroxymethyl	*R*,*S*-phenyl	Cycloheptylmethyl
F		*R*-butyl	*S*-2-butyl	Cyclobutylmethyl
G		*S*-benzyl	*S*-cyclohexyl	3-Methylpentyl
H		*S*-isobutyl	*S*-benzyl	2-Biphenyl-4-yl-ethyl
I			*S*-propyl	4-*Tert*-butyl-cyclohexylmethyl
J				2-(3-Methoxyphenyl)-ethyl
K				2-(4-Isobutylphenyl)-propyl
L				*m*-Tolylethyl
M				*p*-Tolylethyl
N				2-(4-Methoxyphenyl)-ethyl
O				2-(4-Ethoxyphenyl)-ethyl
P				Phenethyl
Q				3-(3,4-Dimethoxyphenyl)-propyl

**Table 2:** Codified analogues and original binding affinities (nM) for FPR1 (p*Ki*1) and FPR2 (p*Ki*2) properties. Values greater than 10,000 were set to p*K* = 4. Compounds declared of interest before application of the SSIR method are specified with asterisks.

Item No.	Analogue	p*Ki*1 ^a^	p*Ki*2 ^b^
1	AAAA	4.000	4.000
2	AABB	4.000	4.000
3	AACA	3.610	4.000
4	ABAC	4.000	4.000
5	ABAA	4.000	4.000
6	ABBC	4.000	4.000
7	ABBB	4.000	4.000
8	ABCC	4.000	4.000
9	BBCA	4.000	2.130 *
10	CBDD	4.000	0.954 *
11	CBCE	3.426	1.079 *
12	CBCF	3.158	0.778 *
13	CCBG	4.000	2.703
14	CCBD	4.000	2.262 *
15	CCBC	4.000	1.839 *
16	CAAC	2.877	1.000 *
17	CAAH	2.550 *	1.322 *
18	CABC	3.527	0.778 *
19	CABH	4.000	1.176 *
20	CACC	2.978	0.699 *
21	CACH	4.000	1.491 *
22	CDAC	3.022	3.176
23	CDAH	2.519 *	2.360 *
24	CDBC	2.858	3.380
25	CDBH	1.663 *	1.845 *
26	CDCC	2.880	2.780
27	CDCH	2.446 *	1.763 *
28	CBAC	2.877	0.903 *
29	CBAH	4.000	1.708 *
30	CBBC	4.000	0.000 *
31	CBBH	4.000	1.322 *
32	CBCC	2.415 *	0.000 *
33	CBCH	2.822	1.041 *
34	BAAC	2.585 *	1.991 *
35	BAAH	3.050	2.243 *
36	BABC	2.639	2.021 *
37	BABH	3.253	1.991 *
38	BACC	3.126	2.212 *
39	BACH	2.943	2.423 *
40	BDAC	2.358 *	4.000
41	BDAH	1.799 *	2.772
42	BDBC	1.954 *	4.000
43	BDBH	0.301 *	2.210 *
44	BDCC	2.985	3.778
45	BDCH	2.675	2.613 *
46	BBAC	3.138	1.869 *
47	BBAH	4.000	2.709
48	BBBC	3.366	1.519 *
49	BBBH	4.000	2.648
50	BBCC	3.543	1.643 *
51	BBCH	4.000	2.657
52	DDBH	0.301 *	3.121
53	EEEA	3.472	4.000
54	DEFA	4.000	4.000
55	DEGA	4.000	4.000
56	DDGA	2.614 *	3.930
57	DEFC	4.000	4.000
58	DEFI	3.266	4.000
59	DDGF	2.888	4.000
60	DFGC	3.368	4.000
61	BEEC	3.291	4.000
62	BEEA	3.349	4.000
63	BEEH	3.102	3.580
64	DEEC	3.740	4.000
65	DEEA	3.504	4.000
66	DEEH	3.177	4.000
67	DEGH	2.901	4.000
68	BGEC	2.941	4.000
69	BGEA	2.766	4.000
70	BGEH	2.083 *	4.000
71	BGGC	2.748	4.000
72	BGGA	2.613 *	4.000
73	BGGH	3.305	4.000
74	BHEC	3.788	3.513
75	BHEA	3.561	3.768
76	BHEH	2.822	4.000
77	BHGC	2.161 *	4.000
78	BHGA	2.666	4.000
79	BHGH	3.054	4.000
80	DGEC	2.672	4.000
81	DGEH	1.716 *	4.000
82	DGGC	2.574 *	4.000
83	DGGA	2.336 *	4.000
84	DGGH	2.775	4.000
85	DHEC	4.000	3.410
86	DHEA	3.226	4.000
87	DHEH	2.772	4.000
88	DHGC	2.238 *	4.000
89	DHGA	2.708	4.000
90	DHGH	2.831	4.000
91	EGEJ	1.176 *	4.000
92	EGEK	0.845 *	4.000
93	EGEH	1.079 *	4.000
94	DGFJ	1.924 *	3.587
95	DGEL	1.301 *	4.000
96	DGEM	1.568 *	4.000
97	DGEJ	1.204 *	4.000
98	DGEN	1.146 *	4.000
99	DGEO	1.863 *	4.000
100	EGEP	0.477 *	4.000
101	EGHQ	2.691	4.000
102	EGIP	0.954 *	4.000
103	EGFP	1.886 *	4.000
104	DDBA	4.000	4.000
105	DDBC	1.447 *	4.000
106	BDBA	4.000	4.000

^a^ The 32 compounds of interest (*Ki*1 ≤ 411) are marked with an asterisk. ^b^ The 32 compounds of interest (*Ki*2 ≤ 410) are marked with an asterisk.

**Table 3:** Area under the receiver operating characteristic (*AU-ROC*) values for several calculations for properties FPR1 and FPR2. The threshold *p_c_* value was set to 0.005 and negation terms were allowed in rules. The number of accepted rules along the loops is given in brackets. For the balanced leave-two-out (BL2O) cross-validation process, the number of well classified pairs, ties and bad pair rankings encountered along the cycles are indicated between slashes. See text for more details.

Property	Rule Order	Overall Fit	L1O	BL2O
FPR1	1	0.768 (4)	0.761 (6)	0.607 (6) 1408/783/177
	2	0.894 (117)	0.792 (171)	0.788 (174) 1917/96/355
	3	0.890 (960)	0.802 (1379)	0.777 (1433) 1909/2/457
FPR2	1	0.934 (16)	0.933 (18)	0.909 (18) 2106/199/63
	2	0.958 (447)	0.947 (478)	0.948 (485) 2254/2/112
	3	0.967 (3428)	0.950 (3756)	0.947 (3811) 2253/0/115

**Table 4:** List of the 26 most significant rules (*p* < 10^−5.5^) of order 2 for the FPR1 property. The vertical bar stands for the negation operator. Each point stands for the *X* wildcard.

Rule #	Vote	Rule			
1	+1	.	G	.	|C
2	+1	|B	G	.	.
3	+1	.	G	.	|Q
4	+1	.	G	|H	.
5	−1	.	|G	.	|K
6	+1	.	G	.	|D
7	−1	.	|G	|I	.
8	+1	.	G	|C	.
9	+1	.	G	|A	.
10	+1	.	G	.	|F
11	+1	.	G	.	|B
12	+1	.	G	|D	.
13	+1	.	G	|B	.
14	−1	.	|G	.	|J
15	+1	|A	G	.	.
16	−1	.	|G	.	|P
17	+1	|C	G	.	.
18	−1	.	|G	.	|N
19	−1	.	|G	.	|L
20	−1	.	|G	.	|Q
21	+1	.	G	.	|G
22	+1	.	G	.	|I
23	−1	.	|G	.	|M
24	+1	.	G	.	|E
25	−1	.	|G	|H	.
26	−1	.	|G	.	|O

**Table 5:** List of the 31 most significant rules (*p* < 10^−9.2^) of order 2 for the FPR2 property. The vertical bar stands for the negation operator. The points stand for the *X* wildcard.

Rule #	Vote	Rule			
1	−1	|C	|A	.	.
2	−1	|C	.	|C	.
3	+1	C	.	.	|G
4	+1	|D	.	|E	.
5	+1	|D	|G	.	.
6	+1	C	|D	.	.
7	+1	C	.	.	|M
8	+1	C	.	.	|N
9	+1	C	.	.	|O
10	+1	C	|G	.	.
11	−1	|C	.	.	|D
12	+1	C	.	|E	.
13	+1	C	.	.	|P
14	+1	C	|H	.	.
15	−1	|C	.	.	|E
16	+1	C	.	|G	.
17	+1	C	.	.	|A
18	+1	C	.	.	|Q
19	−1	|C	.	.	|G
20	+1	C	.	|H	.
21	+1	C	.	|F	.
22	+1	C	.	.	|B
23	−1	|C	|C	.	.
24	+1	C	.	|I	.
25	+1	C	.	.	|J
26	+1	C	|F	.	.
27	+1	C	.	.	|K
28	+1	C	.	.	|I
29	+1	C	.	.	|L
30	+1	C	|E	.	.
31	−1	|C	.	|D	.

## Appendix

### More Algebra among Rules

Special attention has to be given on particular algebraic issues related to the rules treatment. A rule or variable *AB* stands for the simultaneous presence of substituents *A* and *B* at each respective site. This can be stated using the logical and symbol (˄):

AB≡AX∧XB

which could be written in other alternative forms as the intersection of two sets {*AX*} ∩ {*XB*} or simply as *AX* ∩ *XB*. Note that the use of the wildcard *X* is necessary in order to specify without ambiguity the position of each residue.

As seen in the main text, the negative or complementary operator just prevents the use of a particular residue, say *A*, in a specified site, as in $\bar{A}X$. This rule is equivalent to the set {*XX*}∖{*AX*} or to the Cartesian product {*B*,*C*,*D*,...} × {*X*}. The name complementary comes from the set theory because, given a rule, its negation defines the set of elements that are not encompassed by that rule, the complementary set.

The negative operator can be also formulated in terms of a logical not operator (symbol ¬): $\bar{A}\equiv \neg A$. The complementary operator can embrace two or more sites at the same time as in the rule $\bar{AB}$. This rule involves the inclusive or operator (˅):

(A1) $$\bar{AB}\equiv \neg \left(AB\right)\equiv \neg \left(AX\wedge XB\right)\equiv \neg \left(AX\right)\vee \neg \left(XB\right)\equiv \left(\neg A\right)X\vee X\left(\neg B\right)\equiv \bar{A}X\vee X\bar{B}$$

or, in terms of sets notation,

(A2) $$\bar{AB}\equiv \neg \left(AB\right)\equiv \neg \left(AX\cap XB\right)\equiv \neg \left(AX\right)\cup \neg \left(XB\right)\equiv \left(\neg A\right)X\cup X\left(\neg B\right)\equiv \bar{A}X\cup X\bar{B}$$

This rule defines the set of molecules that avoids the simultaneous combination of substituents *A* and *B* at positions 1 and 2, respectively. Note that this definition condenses molecules like *AC* or *DB*. In other words, the use of substituents *A* and *B* in sites 1 and 2 is allowed unless the use is *simultaneous*. As a consequence, it is worthy to note that

$$\bar{AB}\neq \bar{A}\bar{B}$$

because, as seen in the main text in Equation (5), $\bar{A}\bar{B}\equiv \bar{A}X\cap X\bar{B}$ which is not the same as (A.2). Obviously, the general application of the negative operator will lead to the construction of complicated rules that had not been taken into account in this work.

In terms of the strict application of the set theory, one could think that the negative or complementary of the wildcard *X* should be the null set, $\bar{X}=\neg X=\emptyset$. However, this is not because in SSIR context the wildcard does not stand for *all* the substituents but rather for *any* substituent, a more naïve concept. In fact, in Equations (A1) or (A2) it has been used the convention that, formally, the symbol *X* is transparent to the negation operator: $\bar{X}\equiv \neg X\equiv X$. A rule is idempotent under the double negation. In general, $\bar{\bar{A}}=\neg \bar{A}=\neg \left(\neg A\right)=A$. A full negation is generated when the negative operator acts over the entire rule, as in

$$\neg \left(ABXX\right)=\bar{ABXX}=\bar{AB}XX=\bar{A}XXX\cup X\bar{B}XX$$

or

$$\neg \left(A\bar{B}XX\right)=\bar{A\bar{B}XX}=\bar{A\bar{B}}XX=\bar{A}XXX\cup XBXX$$

where in the last case the idempotency relation has been used among *B*.

One must distinguish between the full negation ¬(*XAXA*) and a sequence of partial negations, as in *X*(¬*A*)*X*(¬*A*). The first rule stands for $\bar{XAXA}$ (involving the $\bar{AA}$ concept) whereas the second one is $X\bar{A}X\bar{A}$ (involving the $\bar{A}\bar{A}$ term). This shows how the linear notation of the rules can lead to a problem of interpretation. For instance, one thing is to declare the set $X\bar{A}B\bar{A}$ understood as $X\bar{A}XX\cap XXBX\cap XXX\bar{A}$ and another non-equivalent set is being defined if the rule is understood as $X\bar{AXA}\cap XXBX=\left(X\bar{A}XX\cup XAX\bar{A}\right)\cap XXBX$. In other words, if the molecular residues where re-labeled and, instead, the intrinsic 1-2-3-4 order in variables (*i.e.*, molecular library expanded as *R*_1_ × *R*_2_ × *R*_3_ × *R*_4_) was changed by 1-2-4-3 (codifying the generation of the same library as *R*_1_ × *R*_2_ × *R*_4_ × *R*_3_) the notation changes. In this last case, the first interpretation of the original $X\bar{A}B\bar{A}$ rule will turn to be $X\bar{A}\bar{A}B$, whereas the second interpretation, under relabeling, is now $X\bar{AA}B$. The ambiguities that can arise in certain situations (mainly when the relevant residues are not juxtaposed when writing the rule) can be solved by means of the use of special graphical links indicating which residues stand below the same negation operator. For instance, the second interpretation above would require a special notation like $X\underset{⎵}{\bar{A}B\bar{A}}$.

## References

[1] Kolpak J., Connolly P.J., Lobanov V.S., Agrafiotis D.K. (2009). Enhanced SAR maps: Expanding the data rendering capabilities of a popular medicinal chemistry tool. J. Chem. Inf. Model..

[2] Wassermann A.M., Haebel P., Weskamp N., Bajorath J. (2012). SAR Matrices: Automated extraction of information-rich SAR tables from large compound data sets. J. Chem. Inf. Model..

[3] Agrafiotis D.K., Wiener J.J.M., Skalkin A., Kolpak J. (2011). Single *R*-group polymorphisms (SRPs) and *R*-cliffs: An intuitive framework for analyzing and visualizing activity cliffs in a single analog series. J. Chem. Inf. Model..

[4] Duffy B.C., Zhu L., Decornez H., Kitchen D.B. (2012). Early phase drug discovery: Cheminformatics and computational techniques in identifying lead series. Bioorg. Med. Chem..

[5] Medina-Franco J.L., Edwards B.S., Pinilla C., Appel J.R., Giulianotti M.A., Santos R.G., Yongye A.B., Sklar L.A., Houghten R.A. (2013). Rapid scanning structure-activity relationships in combinatorial data sets: Identification of activity switches. J. Chem. Inf. Model..

[6] Monroc S., Badosa E., Besalú E., Planas M., Bardají E., Montesinos E., Feliu L. (2006). Improvement of cyclic decapeptides against plant pathogenic bacteria using a combinatorial chemistry approach. Peptides.

[7] Badosa E., Ferre R., Planas M., Feliu L., Besalú E., Cabrefiga J., Bardají E., Montesinos E. (2007). A library of linear undecapeptides with bactericidal activity against phytopathogenic bacteria. Peptides.

[8] Feliu L., Oliveras G., Cirac A.D., Besalú E., Rosés C., Colomer R., Bardají E., Planas M., Puig T. (2010). Antimicrobial cyclic decapeptides with anticancer activity. Peptides.

[9] Eriksson L., Johansson E., Kettaneh-Wold N., Wikström C., Wold S. (2000). Design of Experiments. Principles and Applications.

[10] Egan J.P. (1975). Signal Detection Theory and ROC Analysis.

[11] Besalú E., De Julián Ortiz J.V., Pogliani L., Putz M.V. (2010). On Plots in QSAR/QSPR Methodologies. Quantum Frontiers of Atoms and Molecules.

[12] Forlay-Frick P., van Gyseghem E., Héberger K., Vander Heyden Y. (2005). Selection of orthogonal chromatographic systems based on parametric and non-parametric statistical tests. Anal. Chim. Acta.

[13] Mason S.J., Graham N.E. (2002). Areas beneath the relative operating characteristics (ROC) and relative operating levels (ROL) curves: Statistical significance and interpretation. Q. J. R. Meteorol. Soc..

[14] Jin Cho S., Zheng W., Tropsha A. (1998). Rational combinatorial library design. 2. Rational design of targeted combinatorial peptide libraries using chemical similarity probe and the inverse QSAR approaches. J. Chem. Inf. Comput. Sci..

[15] Afshar M., Lanoue A., Sallantin J. (2007). Multiobjective/multicriteria optimization and decision support in drug discovery. Comprehens. Med. Chem. II.

[16] King R.D., Muggleton S., Lewis R.A., Sternberg M.J.E. (1992). Drug design by machine learning: The use of inductive logic programming to model the structure-activity relationships of trimethoprim analogues binding to dihydrofolate reductase. Proc. Natl. Acad. Sci. USA.

[17] Carbó R., Besalú E. (1993). Nested summation symbols and perturbation theory. J. Math. Chem..

[18] Besalú E., Carbó R. (1994). Generalized Rayleigh-Schrödinger perturbation theory in Matrix form. J. Math. Chem..

[19] Carbó R., Besalú E. (1994). Definition, mathematical examples and quantum chemical applications of nested summation symbols and logical kronecker deltas. Comput. Chem..

[20] Carbó R., Besalú E., Defranceschi M., Ellinger Y. (1996). Strategies and Applications in Quantum Chemistry: From Astrophysics to Molecular Engineering Part 2.

[21] Besalú E., Ponec R., de Julián-Ortiz J.V. (2003). Virtual generation of agents against mycobacterium tuberculosis: A QSAR study. Mol. Divers..

[22] Barroso J.M., Besalú E. (2005). Design of experiments applied to QSAR: Ranking a set of compounds and establishing a statistical significance test. Theochemistry.

[23] Yan S.F., Asatryan H., Li J., Zhou Y. (2005). Novel statistical approach for primary high-throughput screening hit selection. J. Chem. Inf. Model..

[24] Besalú E., Vera l. (2008). Internal Test Sets (ITS) Method: A new cross-validation technique to assess the predictive capability of QSAR models. Application to a benchmark set of steroids. J. Chil. Chem. Soc..

[25] De Julián-Ortiz J.V., Besalú E., García-Domenech R. (2003). True prediction by consensus for small sets of cyclooxigenase-2 inhibitors. Indian J. Chem. A.

[26] García-Domenech R., de Julián-Ortiz J.V., Besalú E. (2006). True prediction of lowest observed adverse effect levels. Mol. Divers..

